# Suture anchor fixation strength with or without augmentation in osteopenic and severely osteoporotic bones in rotator cuff repair: a biomechanical study on polyurethane foam model

**DOI:** 10.1186/1749-799X-9-48

**Published:** 2014-08-22

**Authors:** Mehmet Serhan Er, Levent Altinel, Mehmet Eroglu, Ozgur Verim, Teyfik Demir, Halil Atmaca

**Affiliations:** 1 Department of Orthopaedics and Traumatology, Faculty of Medicine, Akdeniz University, Antalya 07985, Turkey; 2 Department of Orthopaedics and Traumatology, Faculty of Medicine, Afyon Kocatepe University, Afyonkarahisar 03200, Turkey; 3 Department of Mechanical Engineering, Faculty of Technology, Afyon Kocatepe University, Afyonkarahisar 03200, Turkey; 4 Department of Biomedical Engineering, TOBB University of Economics and Technology, Ankara 06520, Turkey

**Keywords:** Suture anchor, Pullout, Osteoporosis, Rotator cuff repair, Tricalcium phosphate cement, Bone

## Abstract

**Background:**

The purpose of the present study was to compare the results of various types of anchor applications with or without augmentation in both osteopenic and severely osteoporotic bone models.

**Methods:**

Two different types of suture anchors were tested in severely osteoporotic (SOP) and osteopenic polyurethane (PU) foam blocks using an established protocol. An Instron machine applied static loading parallel to the axis of insertion until failure, and the mean anchor failure strengths were calculated. The mode of failure (anchor pullout, suture tear) was recorded. The anchors tested included the Corkscrew® (CS) (Arthrex Inc., Naples, FL, USA) (without augmentation, polymethylmethacrylate (PMMA)-augmented, and bioabsorbable tricalcium phosphate (TCP) cement-augmented) and Corkscrew® FT II (CS FT II) 5.5 mm (without augmentation as used routinely).

**Results:**

The mean failure loads for both SOP and osteopenic PU foam blocks, respectively, were as follows: CS, 16.2 and 212.4 N; CS with TCP, 75.2 and 396 N; CS with PMMA, 101.2 and 528.8 N; CS FT II, 13.8 and 339.8 N.

**Conclusions:**

Augmentation of CS with TCP or PMMA would be essential to SOP bones. In the osteopenic bone model, although anchor fixation augmented with PMMA is the best fixation method, CS augmented with TCP cement or CS FT II without any need for augmentation may also be used as an alternative.

## Background

Rotator cuff tears (RCT) are common causes of shoulder pain and disability, especially in the elderly population with a high prevalence of osteopenia and osteoporosis. Management of these pathologies would require the use of many sophisticated methods (transposition of the intact subscapularis tendon to cover the superior defect, implantation of fascial autograft or allograft tissue, repair of the existing tendon more medially onto the articular surface, latissimus dorsi tendon transfer, free tendon transfer, or simple decompression with debridement of the rotator cuff) [1]. The quality of cuff healing depends on multiple factors including the biomechanical properties of anchors, angle of insertion, anchor design, suture strength as well as the soft tissue, and bone quality. Because the local poor bone quality and systemic osteoporotic changes may compromise the success of RC repair in the elderly and result in suture anchor loosening, impaired tendon healing, and re-rupture of the RC [2], improvement in fixation quality of suture anchors becomes more essential to these patients.

Not only the systemic bone weakness but also the subchondral bone cysts in the proximal humerus due to chronic cuff tear can lead to poor bone quality and weaker fixation strength [3,4]. Before placing a suture anchor, the surgeon should decide which anchoring position will allow the most anatomic repair. If the suture anchor fails after it is placed, insertion of it to another site can be problematic because it will be a non-anatomic place and tension at the suture-tendon and anchor-bone interfaces will be increased. Increased tensions at those interfaces bring risks for failure of fixation. During the anchor fixation, those risks may be reduced by bone grafting [5], positioning the anchor subcortically [4], and augmentation of the fixation with polymethylmethacrylate (PMMA) or bioabsorbable tricalcium phosphate (TCP) cement [3,6].

It was previously reported that augmentation of metal suture anchor fixation using bioabsorbable TCP cement and PMMA would increase the pullout strength of suture anchors from osteoporotic bones [3,6,7].

Previous studies about suture anchor strength with or without augmentation were conducted on a unique type of synthetic bone blocks. We aimed to compare the results of various anchor applications with or without augmentation in osteopenic and severely osteoporotic (SOP) bone models and to investigate if various types of suture anchors have different performances in bones with various densities. Do we really need to augment the fixation in osteopenic bones or do the fixation methods that are claimed to be more effective in osteopenic bones also work in SOP bones?

## Materials and methods

### Experimental design overview

In this study, two different types of suture anchors were tested in SOP and osteopenic polyurethane (PU) foam blocks. The study was conducted to compare the pullout strengths of the anchors and decide the strongest fixation tool and method.

### PU foam samples

Two different PU foams that represent SOP and osteopenic bones were used [American Society for Testing and Materials (ASTM) grades 5 and 12, respectively] as testing mediums. The biomechanical properties of the bone models are given in Table 1. Synthetic bone materials are widely used in order to provide a more reliable test medium and uniformity between multiple testing groups unlike cadaveric samples. The ASTM F-1839 states that rigid polyurethane foam is an ideal material for the comparative testing of bone screws and other medical devices and instruments [8,9].

**Table 1:** Biomechanical properties of severely osteoporotic and osteopenic bone models

**Density**		**Compressive strength**	**Compressive modulus**
**(pcf)**	**(g/cc)**	**(MPa)**	**(MPa)**
7.5	0.12	0.28	7.5
20.2	0.324	8.4	20.2

**Figure 1:**
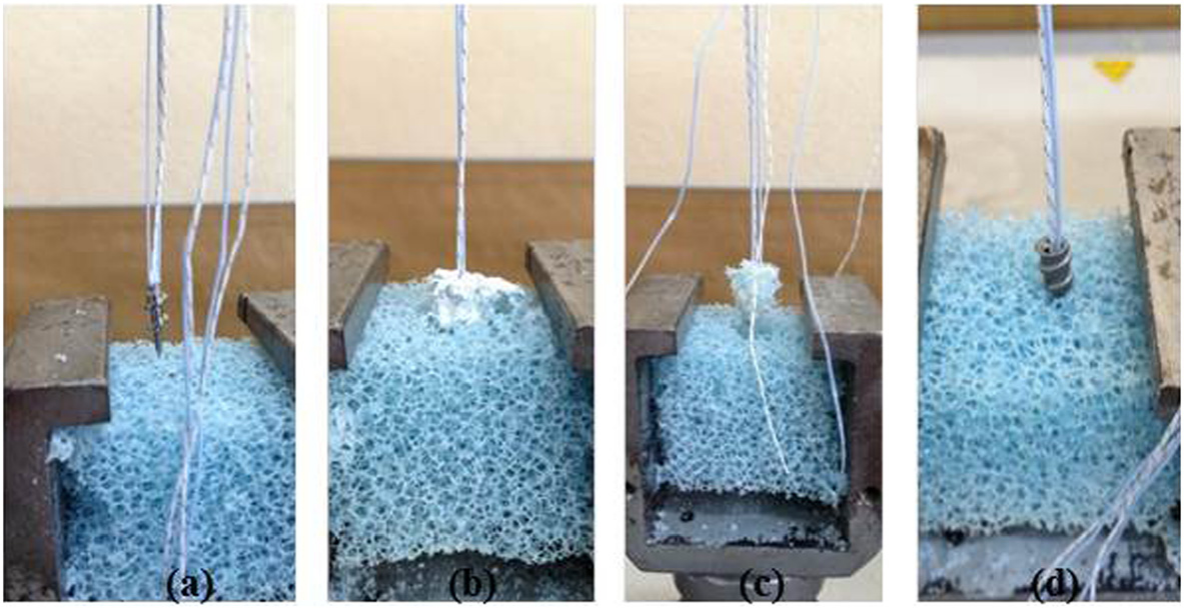
**Tested specimens on severely osteoporotic polyurethane foam block. (a)** Corkscrew®. **(b)** TCP-augmented Corkscrew®. **(c)** PMMA-augmented Corkscrew®. **(d)** Corkscrew® FT II.

**Figure 2:**
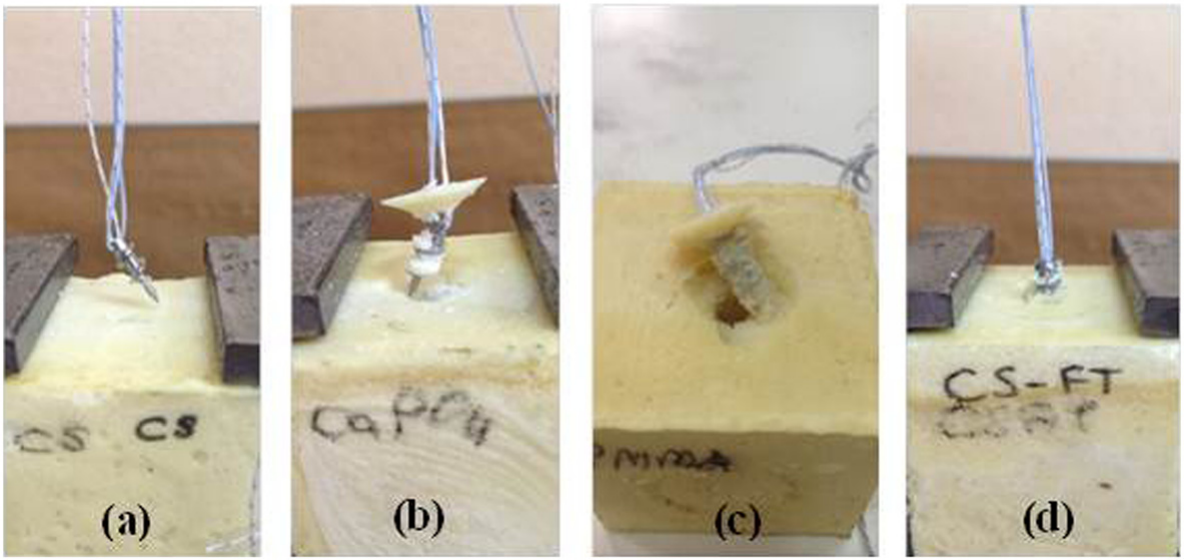
**Tested specimens on osteopenic PU foam block. (a)** Corkscrew®. **(b)** TCP-augmented Corkscrew®. **(c)** PMMA-augmented Corkscrew®. **(d)** Corkscrew® FT II.

SOP samples (Figure 1) were supplied as blocks (65 mm × 50 mm × 50 mm) from Sawbones® Europe AB (Malmö, Sweden). Osteopenic blocks (60 mm × 60 mm × 60 mm) (Figure 2) were produced by the Department of Biomedical Engineering (TOBB University of Economics and Technology). Regular use of the latter foam blocks was approved previously [10].

### Suture anchors

**Figure 3:**
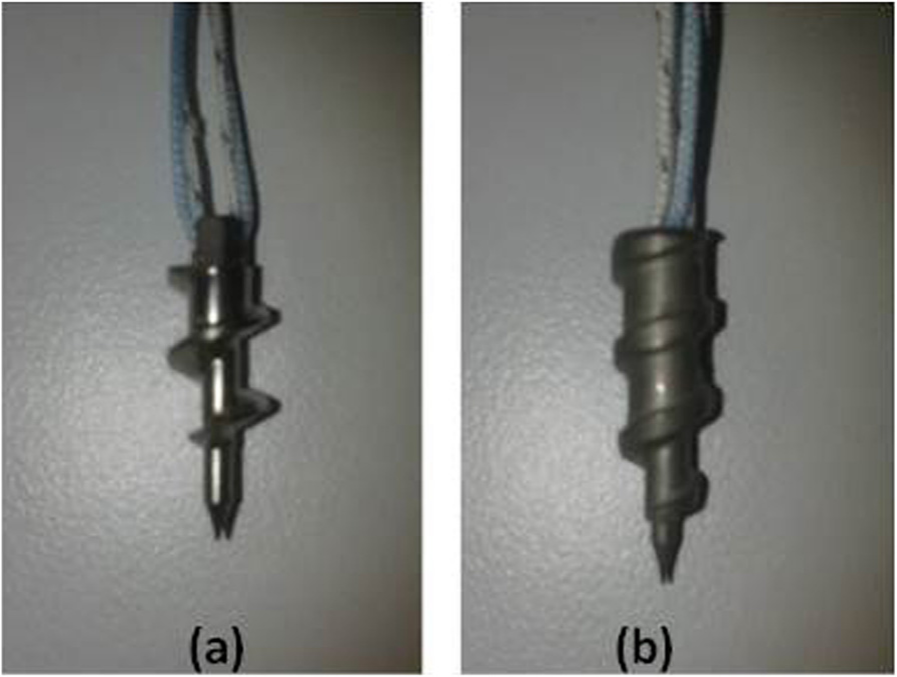
**Suture anchors. (a)** Corkscrew®. **(b)** Corkscrew® FT II.

Two types of suture anchor (Figure 3) were investigated: Corkscrew® (5.5 mm × 15 mm) and Corkscrew® FT II (5.5 mm × 16.3 mm) (Arthrex, Naples, FL, USA).

Corkscrew® (CS) is not fully threaded with a 3-mm part engaged in the applicator, and it has two strands of FiberWire® (Arthrex, Naples, FL, USA). Although Corkscrew® FT II (CS FT II) was designed to maximize fixation in cortical bone, it was tested without any augmentation because in clinical practice, you cannot always manage to find enough cortical support especially in osteoporotic bones. This anchor is fully threaded and has thicker threads to increase the pullout strength when compared to CS.

### Anchor pullout testing

Holes for anchor insertion with augmentation of TCP or PMMA were prepared perpendicular to the surface of the bone model via a Jamshidi needle (CareFusion, San Diego, CA, USA), as previously described, with a depth of 20 mm in all blocks [3].

CS was tested under three conditions: without augmentation, PMMA-augmented, and TCP-augmented. For those anchors augmented with TCP (Callos® Inject, Acumed LLC, Hillsboro, OR, USA), 1.5 to 2.0 mL of material was injected by hand with the use of a syringe-like applicator device available with the TCP cement; a gentle backfill technique along the entire length of the pilot hole just before screw placement was performed. For those anchors applied with PMMA (Surgical Simplex® P, Stryker International, Limerick, Ireland), the cement was prepared manually and inserted as soon as the cement was mixed until homogeneous, immediately placed into a 20-mL syringe while runny, and attached to a Jamshidi needle. PMMA was applied to the pilot holes by using the similar amount and backfill technique as performed with TCP. After application of TCP and PMMA, anchors were inserted to whole length. All procedures were conducted at 24°C and the humidity was 50%.

**Figure 4:**
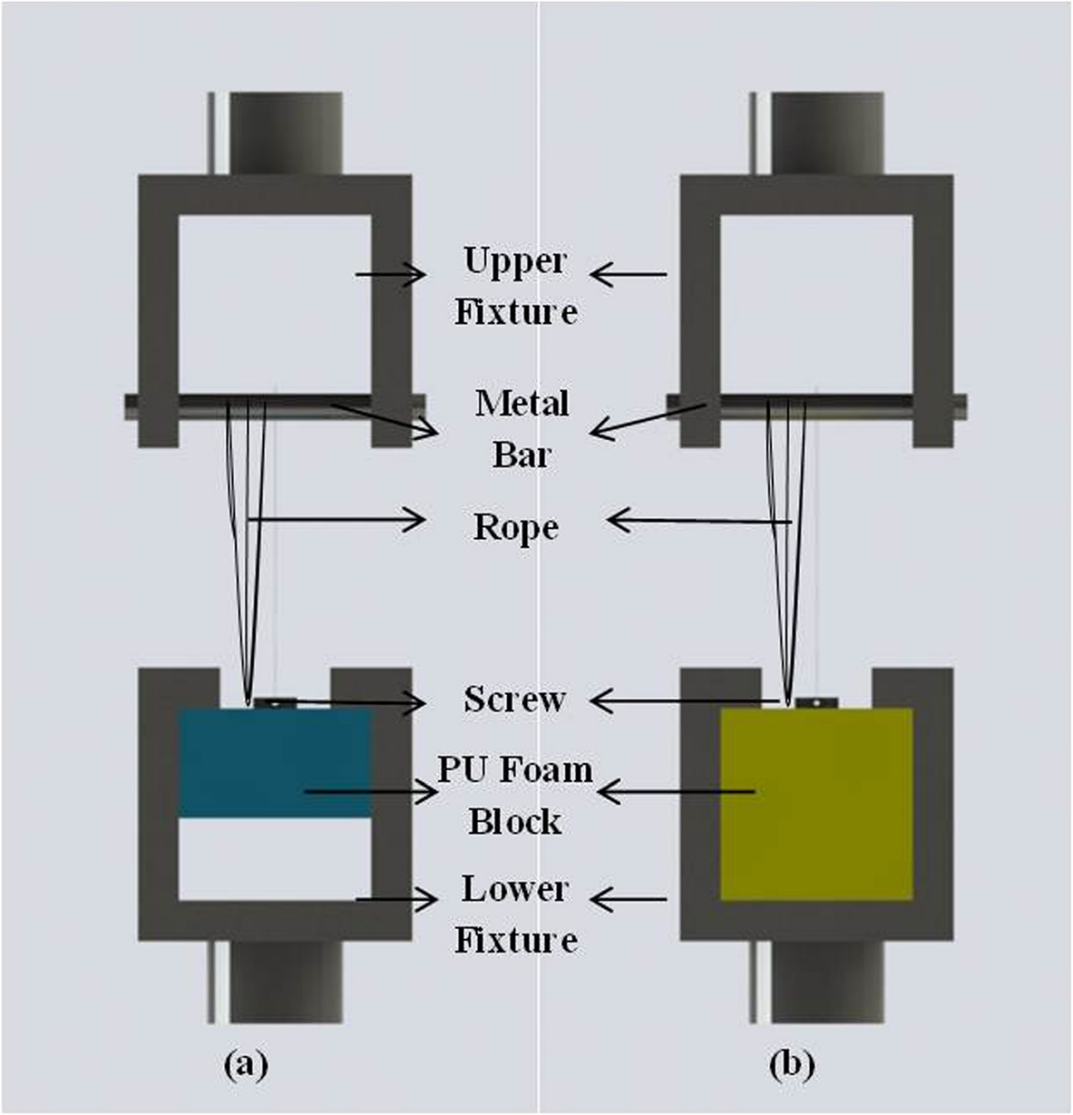
**Test setup. (a)** Severely osteoporotic. **(b)** Osteopenic.

Although in some of the previous studies [2,6,11] sutures of the anchors were replaced with steel wires, we preferred to perform the tests in the original states of the anchors (two strands of #2 FiberWire®). Because we do not use steel wires in clinical practice, we believe that it is more reasonable to assess the anchors without replacing wires to simulate what happens *in vivo*. Besides, the eyelet of CS FT II was made of fiber wire, and if we had replaced it with steel wires, the eyelet would break down against the wire.Bone model blocks were secured to an Instron testing machine using clamping devices. One end of the suture was inserted in the screw's eyelet, and then this end was tied up with the other end of the suture on the metal bar, which was inserted in the upper fixture of the test setup (Figure 4). The length of the suture loop was 100 mm in all tests. The sutures were preloaded with 2 N. To minimize the risk of suture tear, a cylindrical metal bar was used.

Instron 3300 testing machine (Instron Corp., Norwood, MA, USA) was used to pull out the anchors axially, and load versus displacement plots were recorded by its computer software. Axial tension was applied with 25 mm/min constant crosshead speed, and yield load was determined with 0.002 offset methods. For each group, five tests were performed. Failure criteria to stop the test were pulling out of the anchors with peak load from the PU foam blocks or tear of sutures.

All designed anchor systems were tested in accordance with ASTM F543 (ref.11 ASTM Standards, F543-07. Standard specification and test methods for metallic medical bone screws, 2002). Although the cyclic testing is closer to the clinical case, the pullout of the anchors is more prone to occur in the earlier postoperative period. So we applied static pullout tests in all four conditions.

### Statistical analysis

Statistical analyses were performed by using SPSS 15.0 (SPSS Inc, Chicago, IL, USA). Differences in mechanical performance between different anchor constructs were analyzed by using analysis of variance (ANOVA). In order to understand which construct caused differences, data were analyzed by using *post hoc* Bonferroni. Statistical significance was defined as *p* < 0.05.

## Results

Pullout results of constructs are given in Table 2. It was seen that TCP augmentation increased the pullout strength 364% on SOP samples and 86% on osteopenic samples when compared with CS with no cement. On the other hand, PMMA augmentation increased the pullout strength 524% on SOP blocks and 148% on osteopenic blocks when compared with the application of CS without cement. The pullout strength of PMMA augmentation was 34% greater on SOP blocks and 33% greater on osteopenic blocks when compared with TCP augmentation. These differences between CS + PMMA and CS + TCP were statistically insignificant in the SOP model while significant in the osteopenic bone model (Tables 3 and 4).

**Table 2:** Summary of results for both osteopenic and severely osteoporotic bone models

	**Severely osteoporotic**		**Osteopenic**	
	**Ultimate failure load (N)**	**Mode of failure**	**Ultimate failure load (N)**	**Mode of failure**
CS	16.2 ± 3.8	Foam	212.4 ± 64.2	Suture break
CS + PMMA	101.2 ± 33.3	Foam	528.8 ± 45	Suture break
CS FT II	13.8 ± 2.4	Foam	339.8 ± 48	Suture break
CS + TCP	75.2 ± 25.1	Foam	396 ± 52.7	Suture break

**Table 3:** Statistical results of anchor fixations on the severely osteoporotic bone model

	**CS**	**CS + PMMA**	**CS FT II**
CS + PMMA	0.000		
CS FT II	1.000	0.000	
CS + TCP	0.002	0.407	0.002

**Table 4:** Statistical results of anchor fixations on the osteopenic bone model

	**CS**	**CS + PMMA**	**CS FT II**
CS + PMMA	0.000		
CS FT II	0.009	0.000	
CS + TCP	0.000	0.007	0.678

When the pullout strength of CS FT II values were compared with CS, it was seen that CS FT II exhibited 14% lower pullout strength on SOP blocks and 59% higher pullout strength on osteopenic blocks. CS FT II provided statistically significant augmentation in the osteopenic group (*p* = 0.009) (Tables 3 and 4).

The severely osteoporotic PU foam block had a porous structure, so the failure always occurred in the bone-anchor interface; on the other hand, on osteopenic PU foam blocks, mostly, the suture (two strands of #2 FiberWire®) was torn. Tear of the suture showed that anchors can resist higher loads than the suture can.

## Discussion

The quality of rotator cuff repair depends on the reliability of the mechanical strength of the construct and is determined by the weakest link in the chain. Compromised bone quality in the greater tuberosity (GT) due to systemic osteoporosis, immobilization after chronic RCT, subchondral bone cysts caused by chronic impingement, and prior surgery forms the weakest link in this chain [4,6,11,12]. The cancellous part of the GT rather than the cortical part is far more altered in these situations [4].

This is the first study to compare the effects of TCP and PMMA cements in the augmentation of suture anchor fixation on osteopenic and SOP bone models. While the best results for osteopenic bone models were provided by PMMA-augmented CS, both the TCP- and PMMA-augmented CS provided similar results on SOP bone models. These results show that PMMA has a superior effect in increasing pullout strength especially for osteopenic bones. Although CS FT II was designed to obtain higher pullout strength values, PMMA-augmented CS exhibited better test results.

**Figure 5:**
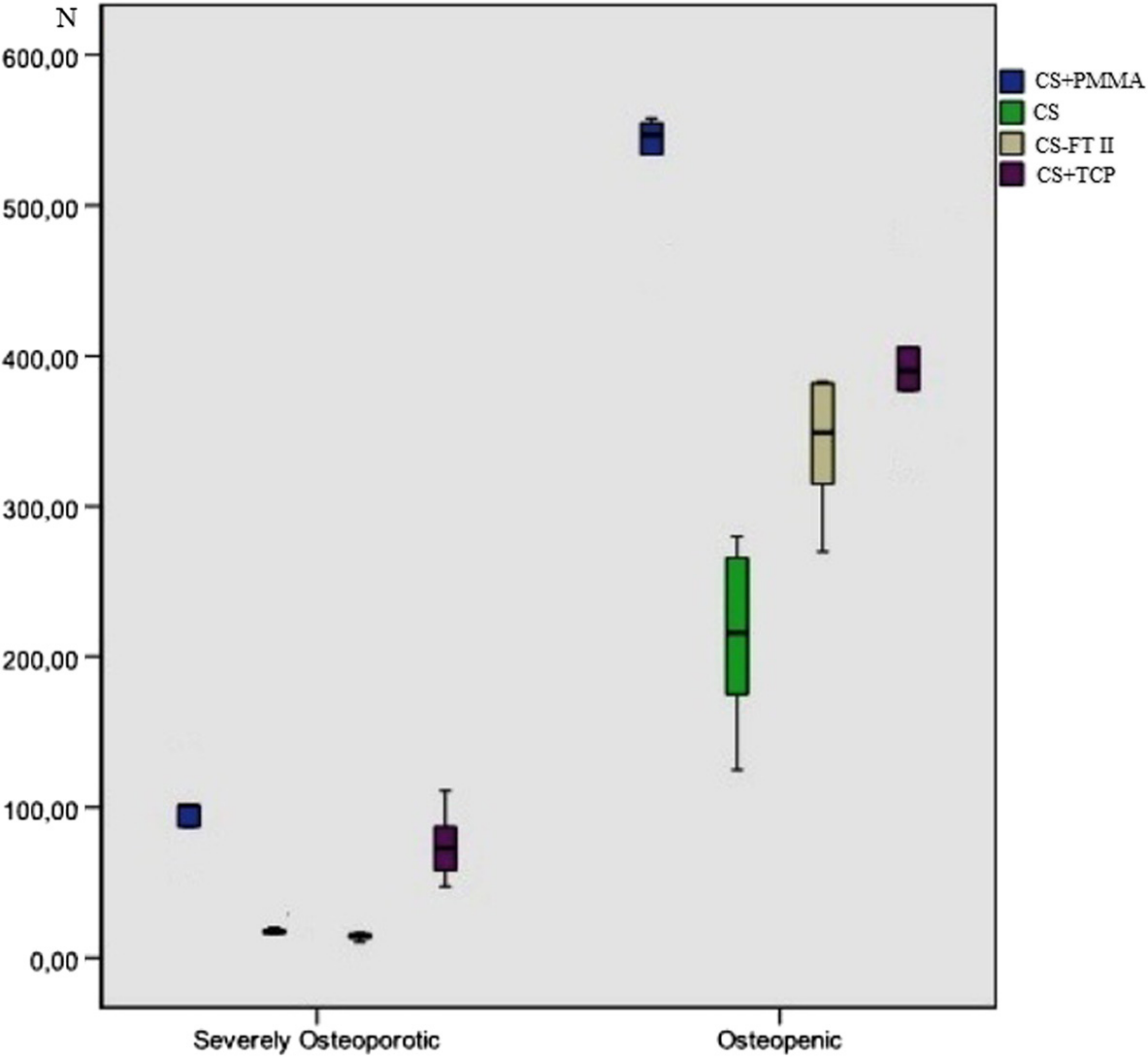
Box plot revealing the results of the pullout tests on the severely osteoporotic and osteopenic bone models.

We searched if the obtained values for pullout strength were high enough to resist failure in the early postoperative period or when a probable failure would occur. For massive tears involving the supraspinatus and infraspinatus tendons, the optimal postoperative shoulder immobilization postures are with the humerus elevated and externally rotated in changing angles according to the size of the defect in the rotator cuff, in order to minimize passive tension in the repaired tendon(s) during the immediate postoperative period and improve the potential for healing [13]. The test results, with the exception of CS and CS FT II pullout tests on SOP bone models, are above the tensile forces of these tendons with the postoperative shoulder immobilization postures [13] (Figure 5).

Barber et al. [14] concluded in their study that there is no correlation with the pullout strength of the screw type anchor and bone mineral density (BMD); however, other studies trying to characterize the bone architecture of the GT and investigating the correlation between pullout strength and BMD in different locations suggested that the bone density varies between individuals but is highest at the posteromedial aspect of the GT [4,12] and the pullout strength of anchor fixations increases with BMD [2,11].

Researchers have investigated whether this emerged problem can be solved by using PMMA or TCP cement augmentation techniques as previously used in fracture fixation and spine surgery [15-18]. In their cadaveric study in which the specimens were not osteoporotic, Oshtory et al. [3] reported an increase of 29% in the pullout strength of suture anchor fixation augmented with TCP cement. On the other hand, Giori et al. stated that augmentation of suture anchor fixation with PMMA bone cement whether the suture anchor hole is stripped results in more cycles to failure and greater maximum load compared with non-PMMA-augmented suture anchors in intact bone. They found that the average maximum load carried by the suture anchor was 71% greater for anchors placed in PMMA-augmented holes compared with anchors inserted into intact bone (294 versus 172 N) [6]. Collinge et al. [15] compared TCP and PMMA in augmentation models using 4.5-mm cortical screws inserted into synthetic osteoporotic bone models. They reported that both cements perform mechanically similar as a screw augmentation material.

PU foam bone models, which are homogeneous, simulate the biomechanical properties of native bone [19], and unlike stripped or drilled holes in cadaveric bones, they eliminate the possibility of encountering a healthy trabecular structure around the anchor that PMMA would interdigitate. The average pullout forces that we report in this study for the SOP model are smaller than those reported by both Meyer et al. and Giori et al., probably because both stripped holes (the holes created by the pullout of a suture anchor) [6] and drilled holes in another study [4] had healthy cancellous bone surrounding these holes so that PMMA would interdigitate. We also used original sutures of anchors instead of steel wires and so preferred to test their original status not to eliminate tear of the suture as a mode of failure.

Although the average pullout forces of PMMA-augmented anchors are greater than those of TCP-augmented ones, injection of PMMA into a suture anchor hole without spilling out of the hole is technically demanding when performing arthroscopically and it should be done through a mini-open approach to avoid the spilling out of PMMA particles in the joint space [6]. The cement would not be so runny to avoid leakage from the hole injected in. Otherwise, the cement particles may act as foreign bodies in the joint space [6]. Within this scope of view, augmentation with TCP cement has an advantage as application of it by a Jamshidi needle could easily be adapted for arthroscopy. Penetration of the cement into the intertrabecular space reaches approximately 0.1 to 2 mm in a normal cancellous bone, but in a SOP bone, further and perhaps unexpected penetration might occur [7], so cemented anchor failure may cause larger defects than non-cemented anchor failure.

Although the cement reaches 90% of its strength after 10 min, it was allowed to cure for 24 h before mechanical testing. This waiting period would not be applicable in clinical practice, because the construct would likely be loaded after surgery before the curing period was completed. It is not clear whether restriction of load bearing by a shoulder immobilizer would eliminate this problem or not. This restriction may reduce the load on the osteopenic construct to subfailure levels but not on the SOP construct.

The static single pullout to failure technique seems as a limitation. But as we focused on the early postoperative failure which would be simulated by static pullout testing, we did not perform a cyclic loading test. Then, we placed the anchor sutures with the angle of 90° to the surface of bone models, not with the angle of 45° to 90° (the so-called dead man's angle) [20], and we used linear pullout axis in line with the anchor's insertion that does not replicate the physiologic pull of the supraspinatus tendon [21]. We thought that this represented a worst-case scenario and would be more suitable to test the anchoring capacity of the suture anchor. Moreover, when an anchor is tested by pulling out axially, it can be thought that failure would occur at higher values in physiological conditions than the measured ones. However, RC repairs made with the screw-in suture anchor inserted at 90° to the superior junction of the greater tuberosity and the humeral head articular surface provided better soft tissue fixation stability than repairs made with the anchors inserted at the dead man's angle of 45° [20]. We used a fully cancellous bone model to simulate the worst condition; however, this would decrease the pullout strength of the fully threaded anchors (such as CS FT II) which mainly anchor the cortical bone. Further studies may explain how it differs to test such anchors in a fully cancellous and a corticocancellous bone model.

## Conclusion

This study showed that in patients with SOP bones, fixation of the ruptured RC with any of these anchors used in this study without augmentation is not clinically applicable as it will most probably fail. Therefore, augmentation with TCP or PMMA would be essential to those patients. For patients with osteopenic bones, although CS augmented with PMMA performed best, CS FT II or CS augmented with TCP may also be used as an alternative choice of fixation for RC repairs. If possible, the bone mineral density may be determined preoperatively to decide if and how augmentation should be performed.

## Competing interests

The authors declare that they have no competing interests.

## Authors' contributions

MSE, LA, and ME participated in the study design, carried out the literature search, performed the data interpretation, and drafted the manuscript. OV and TD performed the biomechanical tests. HA performed the statistical analysis and was involved in revising the manuscript. All authors read and approved the final manuscript.

## Disclosure

The authors or members of their family have not received any financial remuneration related to the subject of the article.
